# A scalable low-cost cGMP process for clinical grade production of the HIV inhibitor 5P12-RANTES in *Pichia pastoris*

**DOI:** 10.1016/j.pep.2015.10.011

**Published:** 2016-03

**Authors:** Fabrice Cerini, Hubert Gaertner, Knut Madden, Ilya Tolstorukov, Scott Brown, Bram Laukens, Nico Callewaert, Jay C. Harner, Anna M. Oommen, John T. Harms, Anthony R. Sump, Robert C. Sealock, Dustin J. Peterson, Scott K. Johnson, Stephan B. Abramson, Michael Meagher, Robin Offord, Oliver Hartley

**Affiliations:** a Department of Pathology and Immunology, Faculty of Medicine, University of Geneva, Geneva, Switzerland; b BioGrammatics Inc., Carlsbad, CA 92011, USA; c Keck Graduate Institute, Claremont, CA, USA; d Biologics Process Development, Inc., Poway, CA 92064, USA; e Department of Medical Protein Research, VIB-UGent, Ghent, Belgium; f Department of Biochemistry and Microbiology, Laboratory for Protein Biochemistry and Biomolecular Engineering, Ghent University, Ghent, Belgium; g University of Nebraska-Lincoln Biological Process Development Facility, College of Engineering, University of Nebraska-Lincoln, Lincoln, NE, USA; h LifeSci Partners, LLC, Vancouver, WA 98686-2811, USA; i The Mintaka Foundation for Medical Research, Geneva, Switzerland

**Keywords:** Pichia, Yeast, Process development, Chemokine, HIV, Microbicide, CCR5, 5P12-RANTES, Biopharmaceutical, cGMP

## Abstract

In the continued absence of an effective anti-HIV vaccine, approximately 2 million new HIV infections occur every year, with over 95% of these in developing countries. Calls have been made for the development of anti-HIV drugs that can be formulated for topical use to prevent HIV transmission during sexual intercourse. Because these drugs are principally destined for use in low-resource regions, achieving production costs that are as low as possible is an absolute requirement. 5P12-RANTES, an analog of the human chemokine protein RANTES/CCL5, is a highly potent HIV entry inhibitor which acts by achieving potent blockade of the principal HIV coreceptor, CCR5. Here we describe the development and optimization of a scalable low-cost production process for 5P12-RANTES based on expression in *Pichia pastoris*. At pilot (150 L) scale, this cGMP compliant process yielded 30 g of clinical grade 5P12-RANTES. As well as providing sufficient material for the first stage of clinical development, this process represents an important step towards achieving production of 5P12-RANTES at a cost and scale appropriate to meet needs for topical HIV prevention worldwide.

## Highlights

• New medicines are required to prevent the spread of HIV/AIDS in low-resource regions.
• 5P12-RANTES, a chemokine analog, is a promising new candidate drug.
• We describe a process for producing clinical grade cGMP 5P12-RANTES in *Pichia pastoris*.
• This is a key step to achieving production at cost and scale appropriate for use worldwide.

## 1 Introduction

There is an urgent need for new products that can be used to stem the spread of HIV/AIDS, particularly in developing countries, where over 95% of the estimated 2 million new infections per year occur [1]. Among the strategies being explored are those centered on the use of topically applied anti-HIV drugs, which can be used to prevent transmission of the virus during sexual intercourse [2].

Blockade of the principal HIV coreceptor, CCR5, is a highly promising strategy for topical HIV prevention [3], [4]. Analogs of the natural chemokine protein ligands of CCR5, including RANTES/CCL5, have potential use as agents for topical HIV prevention [5], [6], [7]. PSC-RANTES was the first chemokine analog to show not only high potency *in vitro* against HIV strains [8] but also full efficacy in a highly stringent and relevant animal model of HIV transmission [5]. However, since it contains non-natural structures that require chemical synthesis steps, PSC-RANTES was considered too expensive to produce for its intended use in resource-poor settings [9].

5P12-RANTES [7] is a next-generation fully recombinant analog which was developed using a phage display approach [10]. In addition to having potency and efficacy comparable to that of PSC-RANTES [7], [11], 5P12-RANTES shows promising stability under storage at elevated temperature and also when exposed to vaginal pH, human cervicovaginal lavage and human semen [12]. Importantly, it has also been shown to present a formidable barrier to the generation of escape mutants [13]. Hence 5P12-RANTES represents a highly promising candidate for development as a topical agent for HIV-prevention.

In order to identify an appropriate recombinant expression system for large scale low cost production of 5P12-RANTES, we focused on yeast secretory expression, which has been shown to be robust and effective for other chemokines [14], [15], [16], including analogs of RANTES/CCL5 [17], [18]. In this study we made use of expression in *Pichia pastoris* (formally classified as *Komagataella phaffii* [19]) to develop and optimize of a low-cost, scalable cGMP-compliant process for production of clinical grade 5P12-RANTES (i.e. >95% purity; endotoxin, host cell protein and host cell DNA levels within regulatory limits).

## 2 Materials and methods

### 2.1 Chemically synthesized reference standard 5P12-RANTES

5P12-RANTES was synthesized as described previously [7] on a modified ABI 430 peptide synthesizer customized to perform Boc chemistry with *in situ* neutralization [20]. The synthesis strategy featured a chemical capping step (to terminate any chains with free amine groups at the end of the coupling step) using acetylglycine at each cycle. After hydrogen fluoride cleavage, crude product was analyzed by reverse-phase HPLC and MALDI mass spectrometry. After preparative scale purification of the desired product, refolding of the protein and formation of disulphide bridges was carried out according to published procedures [21], and the refolded material was verified by reverse-phase HPLC (shorter retention time) and electrospray mass spectrometry (loss of mass units due to oxidation of cysteine thiol group during disulphide bridge formation). A final preparative scale purification was performed on the refolded material, which was then lyophilized prior to use.

### 2.2 Determination of anti-HIV activity

Anti-HIV activities of samples were determined using a CCR5-tropic envelope-dependent cell fusion assay as previously described [7], [8].

### 2.3 Pilot process

#### 2.3.1 Strain for pilot work

A synthetic gene encoding 5P12-RANTES [7] was synthesized (Integrated DNA Technologies) and ligated as an in-frame fusion directly after the KREAEA sequence of the alpha mating factor encoded by the pJAZaMF expression vector [22] (Biogrammatics), which carries the Alcohol Oxidase 1 (AOX1) promoter, allowing inducible expression on methanol. This vector was then linearized at the PmeI site in the AOX1 promoter and transformed into the *P. pastoris* strain Bg08 (Biogrammatics). Zeocin-selected clones were isolated and assessed for expression of 5P12-RANTES.

#### 2.3.2 ELISA assay for quantification of 5P12-RANTES levels

Because of the sequence similarities between 5P12-RANTES and native human RANTES/CCL5, it was possible to set up an ELISA detection assay for 5P12-RANTES using a pair of commercially available antibodies sold as components of a kit for detecting levels of native RANTES/CCL5 (MAB678 for capture, BAF278 for detection, R&D Systems). The assay was performed according to the manufacturer's instructions, except that standard curves were prepared using 5P12-RANTES rather than native CCL5. The assay provided a linear detection range for 5P12-RANTES concentrations between 0.063 ng/mL and 2 ng/mL. Samples of culture supernatant were subjected to a range of dilutions and used as test samples in the ELISA assay, with concentrations of 5P12-RANTES estimated by back-calculating from the resulting absorbance values.

#### 2.3.3 Cation exchange chromatography

Cation exchange chromatography was carried out using a Varian chromatography system on a 11 × 1 cm SP Sepharose Fast Flow column (Amersham Pharmacia) equilibrated with 10 column volumes of 50 mM Bicine buffer pH 7.5. 15 mL of *P. pastoris* culture supernatant was pH adjusted to 8.5 with triethylamine, centrifuged (18000 *g*, 5 min), and loaded in successive 1 mL injections. Separation was achieved by applying a gradient of 0–2 M NaCl in 50 mM Bicine buffer pH 7.5 over 30 min with a flow rate of 1 mL/min.

#### 2.3.4 Reverse phase chromatography (RP-HPLC)

Semi-preparative RP-HPLC was carried out using a Waters 2795 HPLC Separation Module with a 2487 Dual λ Absorbance Detector. The 15 mL eluate peak collected from the cation chromatography step was diluted with 15 mL of 0.1% TFA, acidified with 0.4 mL of glacial acetic acid and passed through 1 mL C18 Sep-Pak cartridges (WAT051910, Waters) that had been pre-equilibrated with 0.1% TFA. Cartridges were then washed with 20 mL of 0.1% TFA and eluted with 4 mL of 0.1% TFA: acetonitrile, 1:1 (v/v). The eluates from the cartridges were pooled, dried, and taken up in 50% acetonitrile/0.1% TFA. 20 μl samples were injected on to a Macherey–Nagel 300 Å μm C8 column (250 × 4 mm) and a gradient (22.5%–49.5% acetonitrile in 0.1% TFA) was applied over 30 min with a flow rate of 0.7 ml/min.

#### 2.3.5 Mass spectrometry

Masses of samples purified by reverse phase HPLC were determined on a Platform LCZ (Micromass) electrospray positive ionization mass spectrometer. Mass spectra were acquired and analyzed using MassLinx software.

### 2.4 Scaled-up process

#### 2.4.1 Strain for cGMP-compliant production of clinical grade material

A fully synthetic construct containing codon-optimized 5P12-RANTES open reading frame (ORF) fused in–frame with the *Saccharomyces cerevisiae* alpha mating factor was synthesized (GeneArt, LifeTechnologies). This construct was flanked with a 5′ EcoRI and a 3′ NotI-restriction site for cloning into the pPpT4αS-expression vector (Graz University of Technology, TU Graz). The final pPpT4αMF-5P12-RANTES expression vector carries a Zeocin^®^ selection marker with 5P12-RANTES-expression under control of the AOX1 promoter. All cloning was done using TSE/BSE-free certified restriction enzymes (New England Biolabs).

The pPpT4αMF-5P12-RANTES vector was PmeI-linearized in the AOX1-promoter prior to electroporation to the *P. pastoris* NRRL Y-11430 strain. Zeocin-selected clones were isolated and assessed for expression of 5P12-RANTES. The best-expressing clone, was taken through a standard cell banking procedure.

#### 2.4.2 Screening for 5P12-RANTES expression

Single clones were used to inoculate 2 mL BMGY medium (1% yeast extract, 2% Soy Peptone, 100 mM potassium phosphate buffer pH 5.5, 1.34% Yeast Nitrogen Base, 1% glycerol) in 24-well deep well plates and incubated at 28 °C and 225 rpm for 48 h. The cells were harvested by centrifugation (265 *g*, 10 min, 4 °C) and taken up in 2 mL unbuffered BMY medium (1% yeast extract, 2% Soy Peptone, 1.34% Yeast Nitrogen Base). Prior to induction, the culture was acidified to pH 3.5 using 1 M citric acid. The cultures were induced with methanol (1% final, v/v) and induction was maintained by spiking the cultures every 8–12 h with an additional 1% MeOH. After induction, the supernatant was collected by centrifugation (265 *g*, 10 min, 4 °C), snap-frozen in liquid nitrogen and kept at −20 °C until further analysis.

#### 2.4.3 In-process detection of 5P12-RANTES using strong cation exchange HPLC

Levels of 5P12-RANTES could be determined directly from fermentation and in-process samples using strong cationic exchange chromatography (SCX). A PolySULFOETHYL A column (PolyLC, Inc; 100 × 4.6 mm) operating with a flow rate of 1 mL/min was equilibrated with Buffer A (50 mM 2-(*N*-morpholino)ethanesulfonic acid (MES), pH 6.4). Following sample application, the following gradient was applied: 2 min–8 min; 0%–30% Buffer B (50 mM MES, 1 M NaCl, pH 6.4), 8 min–40 min 30%–55% Buffer B, 40 min–43 min; 55%–100% Buffer B. Eluted protein was detected by measuring absorbance of at 280 nm. Using this method 5P12-RANTES with the N-terminal glutamine residue cyclized to pyroglutamate elutes after approximately 14 min, and 5P12-RANTES with the N-terminal glutamine residue uncyclized elutes after approximately 15 min. The method has a linear range from 0.25 to 300 μg.

#### 2.4.4 Analytical RP-HPLC for final assessment of purity

Analytical RP-HPLC was performed on an Alliance HPLC system with a 4.6 mm × 150 mm 300 Å 3 μm C8 column (ACE), using mobile phases A (0.1% TFA in water), B (0.0955% TFA/30% acetonitrile in water) and C (0.085% TFA/95% acetonitrile in water). Flow rate was 1 mL/min. Following sample loading, the gradient program shown in Table 1 was applied.

### 2.5 19 L Scale process

#### 2.5.1 Fermentation

A 1 L baffled shake flask containing 300 mL of buffered minimal glycerol yeast extract medium (12.61 g/L glycerol, 10.0 g/L yeast extract, 20.0 g/L soytone, 1.34% YNB, 4 × 10-5% biotin, 100 mM potassium phosphate, pH 6.0) was inoculated with 0.5 mL of the working cell bank and incubated for 24–28 h at 28 °C and 250 rpm until the culture OD_600_ reached ≥ 5.

The shake flask culture was used to inoculate a 19 L NLF fermenter (Bioengineering, Inc) containing 6 L of basal salts medium (0.93 g/L CaSO_4_, 18.2 g/L K_2_SO_4_, 14.9 g/L MgSO_4_·7H_2_O, 4.13 g/L KOH, 40.0 g/L glycerol, 26.7 mL/L of 85% H_3_PO_4_) supplemented with 4.35 mL/L PTM1 salt solution (6 g/L CuSO_4_·5H_2_O, 20.0 g/L ZnCl_2_, 0.08 g/L NaI, 65.0 g/L FeSO_4_·H_2_O, 3.0 g/L MnSO_4_·H_2_O, 0.020 g/L H_3_BO_3_, 0.20 g/L Na_2_MoO_4_·2H_2_O, 0.50 g/L CoCl_2_·6H_2_O, 5 mL/L H_2_SO_4_.

Fermentation was conducted at 30 °C with the airflow fixed at 6 L/min and a pressure set-point of 0.20 bar. During the initial batch phase, pH was maintained at 5.0 using 28–30% ammonium hydroxide and the dissolved oxygen level maintained at 40%. After 18–24 h, glycerol fed-batch growth (feeding with 20 g/L/h glycerol) was initiated for 1 h, then the temperature was reset to 27 °C and the pH allowed to decrease to 3.25 over the course of 1 h. Culture induction was then initiated by adding a 1 g/L bolus of methanol and decreasing the glycerol feed from 20 g/L/h to 0 g/L/h over 3 h. Once the culture had consumed the initial methanol bolus, methanol fed-batch growth was initiated (methanol supplemented with 12 mL/L PTM1 salts and 12 mL/L 5% KFO 673 antifoam provided at an initial rate of 0.027 mL/L/min, increased to 0.066 mL/L/min over the course of 3 h). A ten-step methanol feed profile was then initiated (feed rates shown in Table 2), with induction maintained over 96 h culture.

#### 2.5.2 Harvest by tangential flow filtration

A Model 10 tangential flow filtration (TFF) skid equipped with a 0.93 m^2^ regenerated cellulose 100 kDa MWCO membrane was equilibrated with 2 L of equilibration buffer (25 mM sodium acetate, pH 4.0) supplemented with 300 mM NaCl. Approximately 10.9 L of fermentation broth was pumped into the system and concentrated to 10 L. During filtration, retentate was agitated with an overhead mixer to prevent the cells from settling. Retentate volume was maintained at ∼10 L as the cells were washed with 4 volumes of equilibration buffer. The bulk permeate was collected and saved for further processing.

#### 2.5.3 Preparation of Butyl 650 M load

Approximately 40.6 L of bulk permeate was adjusted to 2.5 M NaCl (via addition of granular NaCl) during mixing over 45 min, with a further 15 min mixing stage appended to ensure that NaCl dissolution was complete. The permeate was than filtered through a 0.2 μm Supor filter (Pall).

#### 2.5.4 Capture of 5P12-RANTES with Butyl 650 M

A 10 cm × 8.5 cm Butyl 650 M column was cleaned with 1 M NaOH for 1 h and equilibrated with equilibration buffer adjusted to 2.5 M NaCl. Filtered permeate was loaded onto the column which was then washed with 5 column volumes equilibration buffer adjusted to 2.5 M NaCl. Elution was performed over 10 column volumes following a linear gradient from 100% equilibration buffer adjusted to 2.5 M NaCl to 100% equilibration buffer. A 10.9 L product fraction was collected.

#### 2.5.5 Capto MMC intermediate chromatography

A 6 cm × 18.3 cm Capto MMC column was cleaned with 1 M NaOH for 1 h. Following equilibration with MMC equilibration buffer (25 mM *N*-cyclohexyl-3-aminopropanesulfonic acid, 25 mM sodium acetate, 25 mM sodium phosphate, pH 4.0), the eluate from the Butyl 650 M chromatography was loaded on to the column, which was then washed with five column volumes of equilibration buffer. Elution of 5P12-RANTES was achieved over a 10 column volume linear gradient starting at 100% MMC equilibration buffer and ending at 100% MMC equilibration buffer supplemented with 2 M NaCl and adjusted to pH 10.0. A product fraction of 7.7 L was collected.

#### 2.5.6 pH adjustment

The Capto MMC product was pH adjusted to pH 4.0 by adding approximately 145 mL 50% acetic acid over 20 min. The pH adjusted product was stored overnight at 4 °C.

#### 2.5.7 Concentration/diafiltration

A tangential flow filtration system consisting of a positive displacement pump (PDL-004-FILT) equipped with a 0.46 m^2^ Ω 3 kDa MWCO flat sheet membrane was equilibrated with 300 mL of diafiltration buffer (100 mM sodium phosphate buffer, pH 6.0). The 7.7 L of pH adjusted Capto MMC product was concentrated to 500 mL, and the retentate volume was maintained at 500 mL during diafiltration (6 volumes of diafiltration buffer), which was carried out at room temperature over 175 min. The final retentate volume was 1.65 L (pH 5.9) after rinsing the system with 3 × 500 mL diafiltration buffer.

#### 2.5.8 Cyclization of the N-terminal glutamine residue

The final retentate from the diafiltration step was diluted to 10.7 L with diafiltration buffer to reach a concentration of ∼1 mg/mL and transferred to a clean NLF fermentation vessel (Bioengineering, Inc), where it was subjected to 4 h incubation at 60 °C, under gentle agitation and N_2_ sparge to maintain dissolved oxygen levels at ≤ 1.0%.

#### 2.5.9 CHT (Ceramic Hydroxyapatite) type I 20 μm polishing chromatography

10.5 L of N-terminal glutamine-cyclized 5P12-RANTES was filtered through a 0.2 μm PVDF filter (Millipore) and slowly added to ∼94.5 L of CHT dilution buffer (50 mM MES, pH 6.5). The diluted filtrate was loaded overnight at 200 mL/min on to a 10 cm × 15 cm CHT type I 20 μm column that had been equilibrated with three column volumes of CHT equilibration buffer (50 mM MES, 5 mM sodium phosphate, pH 6.5). The column was then washed initially with five column volumes CHT equilibration buffer, and subsequently with 100 column volumes of CHT wash buffer (50 mM MES, 5 mM sodium phosphate, 100 mM sodium chloride, pH 6.5). Elution of 5P12-RANTES was achieved over a 30 column volume gradient starting at 100% CHT wash buffer and ending at 100% CHT wash buffer adjusted to 200 mM NaCl. Fractions were analyzed by RP-HPLC for purity, with selected fractions combined to generate a ∼31 L pool of purity >95%, which was stored at 4 °C overnight.

#### 2.5.10 Final concentration/diafiltration and bulking

The tangential flow filtration system used in the concentration/diafiltration was cleaned with 0.5 M NaOH for 1 h, extensively rinsed with posidyne-filtered reverse osmosis water, and equilibrated with 300 mL of final bulking solution (1.7 mM acetic acid pH 4.0). The pooled CHT product was concentrated from 31 L to 400 mL and diafiltered against 8 volumes of final bulking solution. The diafiltrate was then concentrated to the minimum volume of the system (425 mL) and drained. The recovered material was filtered using a 0.2 μm Supor filter (Pall) and stored at −80 °C.

### 2.6 150 L Scale process

#### 2.6.1 Seed train

Two 1 L baffled shake flasks containing 300 mL of buffered minimal glycerol yeast extract medium were inoculated with 0.5 mL of the working cell bank and incubated for 15–30 h at 28 °C and 250 rpm until the culture OD_600_ reached ≥5. 63 mL of pooled shake flask culture was used to inoculate an ALF 903 fermenter (New Brunswick) containing 3.36 L of buffered minimal glycerol yeast extract medium. Fermentation was run for 8–15 h at 30 °C (cascade agitation 200–800 rpm, airflow at 4.2 L/min, dissolved oxygen levels maintained at 40%) until the culture OD_600_ reached ≥ 5.

#### 2.6.2 Fermentation

The entire seed culture was then used to inoculate a 200 L fermenter (Bio Engineering) containing 83 L of basal salts medium supplemented with 362.1 mL of PTM1 salt solution and 83 mL of 5% KFO 673 antifoam. Fermentation was conducted at 30 °C with the airflow fixed at 83 standard liters per minute, agitation at 100–600 rpm. During the initial batch phase, pH was maintained at 5.0 using 20–30% ammonium hydroxide and the dissolved oxygen level maintained at 40%. When glycerol in the medium was exhausted (after 18–24 h fermentation), indicated by a dissolved oxygen spike, glycerol fed-batch growth (feeding a total of 3.75 L 63% (w/v) glycerol containing 12 mL/L PTM1 salt solution over 4 h) was initiated. Glycerol fed-batch consisted of a 1 h feed of 20 g/L/h followed by a 3 h ramp down to 0 g/L/h. The temperature was then reset to 27 °C and the pH allowed to decrease to 3.25 over the course of approximately 1 h. Induction was then initiated by adding a 1 g/L bolus of methanol. Once the culture had consumed this initial methanol bolus, methanol fed-batch growth was initiated (methanol supplemented with 12 mL/L PTM1 salt solution and 12 mL/L 5% KFO 673 antifoam provided at an initial rate of 0.027 mL/L/min, increased to 0.066 mL/L/min over the course of 3 h). The same ten-step methanol feed profile as used in the 19 L scale fermentation was then initiated (Table 2) with the exception that fermentation was halted after 72 h induction.

#### 2.6.3 Harvest by tangential flow filtration

A Model 50 tangential flow filtration (TFF) skid equipped with a 4.64 m^2^ regenerated cellulose 100 kDa MWCO membrane was equilibrated with 20 L of equilibration buffer supplemented with 300 mM sodium chloride. Retentate volume was maintained at 50 L as the cells were washed with 4 volumes of equilibration buffer supplemented with 300 mM sodium chloride. The bulk permeate was collected for further processing.

#### 2.6.4 Preparation of Butyl 650 M load

Bulk permeate was adjusted to 2.5 M NaCl (via addition of granular NaCl) during mixing over 45 min, with a further 15 min mixing stage appended to ensure that NaCl dissolution was complete. The permeate was than 0.2 μm filtered into 200 L barrel bags (Sartorius).

#### 2.6.5 Capture of 5P12-RANTES with Butyl 650 M

A 30 cm × 15 cm Butyl 650 M column was equilibrated with three column volumes of equilibration buffer supplemented with 2.5 M NaCl. The column was loaded with the filtered permeate and washed with five column volumes of equilibration buffer supplemented with 2.5 M NaCl. Elution was performed by passing a linear gradient from 100% 2.5 M NaCl in equilibration buffer to 100% equilibration buffer over ten column volumes, followed by a hold phase (100% equilibration buffer) until completion of elution.

#### 2.6.6 Capto MMC intermediate chromatography

A 30 cm × 15 cm Capto MMC column was equilibrated with three column volumes of MMC equilibration buffer. The eluate from the Butyl 650 M chromatography step was then loaded on to the column, which was then washed with five column volumes of MMC equilibration buffer. Elution of 5P12-RANTES was achieved over a 10 column volume linear gradient starting at 100% MMC equilibration buffer and ending at 100% MMC equilibration buffer supplemented with 2 M NaCl and adjusted to pH 10.0.

#### 2.6.7 Concentration/diafiltration

A Model 10 tangential flow filtration skid equipped with a 4.64 m^2^ regenerated cellulose 3 kDa MWCO membrane was flushed with 5 L of diafiltration buffer, drained and then loaded with the MMC eluate. The retentate was collected after 15-fold concentration and diafiltration against six volumes of diafiltration buffer.

#### 2.6.8 Cyclization of the N-terminal glutamine residue

The diafiltration retentate was diluted with diafiltration buffer to reach a concentration of 1 mg/mL 5P12-RANTES and transferred to a 250 L process tank, where it was subjected to 4 h incubation at 60 °C, under gentle agitation and N_2_ sparge to maintain dissolved oxygen levels at ≤ 1.0%. Material was diluted 20-fold into CHT dilution buffer (50 mM MES, pH 6.5) and 0.2 μm filtered into barrel bags (Sartorius).

#### 2.6.9 CHT (Ceramic Hydroxyapatite) type I 20 μm polishing chromatography

The diluted material was loaded at on to a 45 cm × 11 cm CHT type I 20 μm column that had been equilibrated with three column volumes of CHT equilibration buffer. The column was first washed with five column volumes CHT equilibration buffer, then with 100 column volumes of CHT wash buffer. Elution of 5P12-RANTES was achieved over a 30 column volume linear gradient starting at 100% CHT wash buffer and ending at 100% CHT wash buffer adjusted to 200 mM NaCl, followed by a hold phase (100% CHT wash buffer adjusted to 200 mM NaCl) until elution was complete. Fractions were analyzed by RP-HPLC for purity, with selected fractions combined to generate a pool of purity >95%.

#### 2.6.10 Final concentration/diafiltration and bulking

A Model 10 tangential flow filtration skid equipped with a 2.32 m^2^ regenerated cellulose 3 kDa MWCO membrane was flushed with 2 L of final bulking solution, drained and then loaded with the pooled CHT eluate. The retentate was collected after concentration calculated to yield a final concentration of 8.5 g/L 5P12-RANTES and diafiltration against eight volumes of final bulking solution.

## 3 Results

### 3.1 Pilot expression studies

Zeocin-resistant clones obtained by transforming *P. pastoris* strain Bg08 with the pJAZaMF encoding 5P12-RANTES were screened for 5P12-RANTES expression. A single clone, clone #86 (Mut^+^ phenotype), was selected for pilot expression in a 5 L fermenter.

**Fig. 1:**
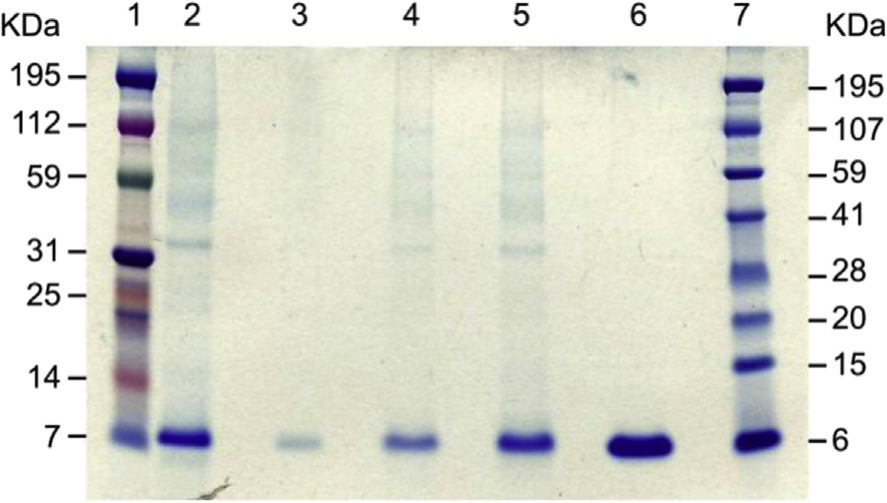
Pilot study of 5P12-RANTES expression by secretion in *P. pastoris*. Coommassie stained SDS-PAGE gel of culture supernatant from an unoptimized 5 L fermentation. Lanes 1 and 7: molecular mass markers; Lane 2: 20 μl unpurified culture supernatant; Lanes 3, 4 and 5: TCA precipitates of larger volumes of culture supernatant corresponding to 220 μl, 450 μl and 900 μl, respectively; Lane 6: chemically synthesized 5P12-RANTES reference standard (4 μg).

The harvested culture supernatant, untreated or following 10% trichloroacetic acid (TCA) precipitation, was analyzed by SDS-PAGE alongside a chemically synthesized reference standard sample of 5P12-RANTES (Fig. 1). The position of the major band from the untreated culture supernatant on the gel with respect to the molecular mass markers and the reference standard sample was consistent with its identity as 5P12-RANTES. Further confirmation was obtained by Western blot using an anti-human RANTES/CCL5 antibody (data not shown). TCA precipitation of increasing volumes of the culture supernatant did not give an increase of band intensities in proportion to the volumes taken, indicating that only a fraction of the total target protein is precipitated under these conditions.

Using an ELISA assay we were able to estimate the concentration of 5P12-RANTES in the crude culture supernatant as 100 mg/L.

### 3.2 Lab-scale purification

**Fig. 2:**
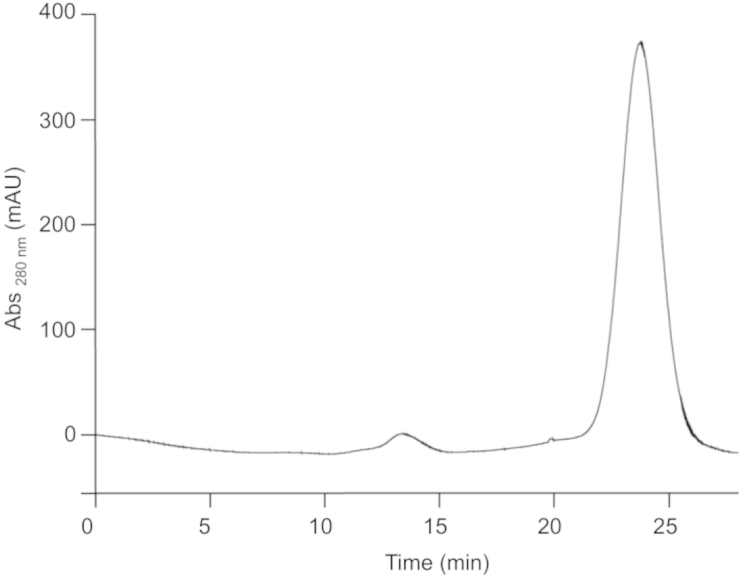
**Semi-preparative pilot purification of crude culture supernatant by cation exchange chromatography**. 15 mL Culture supernatant from the 5 L fermentation was subjected to cation exchange chromatography on an SP Sepharose Fast Flow resin column. The major peak (approx. 24 min) was collected for further analysis. The minor peak (approx. 13 min) corresponded to elution of strongly pigmented material present in the culture supernatant.

We next purified the material for further characterization using a two-step procedure. The first step, cation exchange chromatography, takes advantage of the high isoelectric point of 5P12-RANTES (predicted value 9.4, based on primary sequence using the *Compute pI/Mw* tool (www.expasy.org)). Cation-exchange chromatography of a 15 mL sample of culture supernatant resulted in the elution of a single major peak (Fig. 2).

**Fig. 3:**
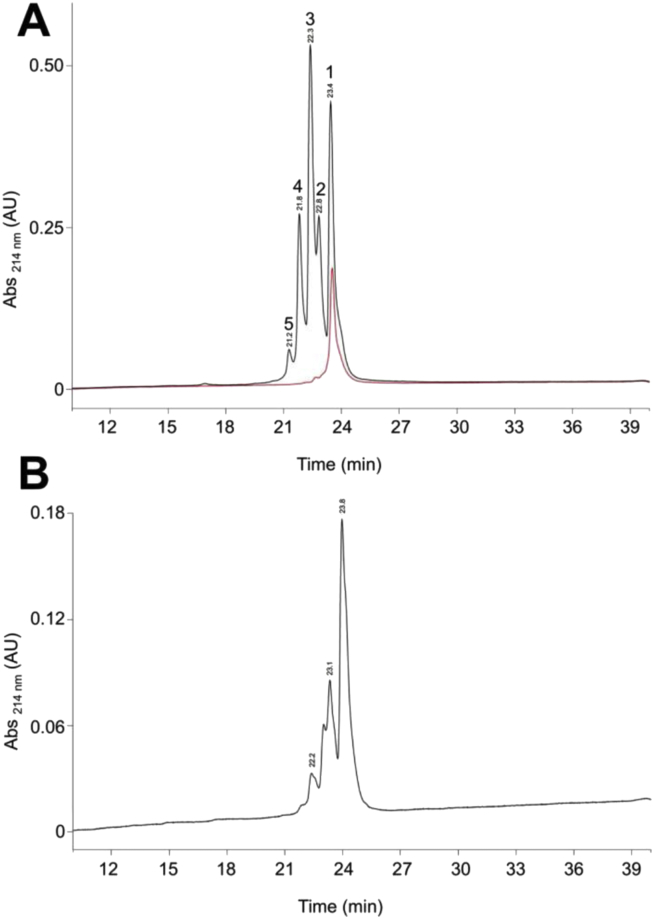
**RP-HPLC purification of 5P12-RANTES**. Pooled material eluted from the previous cation chromatography step was subjected to semi-preparative RP-HPLC purification. **A**. Untreated pooled eluate (red trace: a chemically synthesized standard). **B**. Pooled eluate following treatment to promote cyclization of the N-terminal glutamine residue (pH 8.5, 37 °C, 64 h). Retention times in minutes are indicated above each peak. (For interpretation of the references to colour in this figure legend, the reader is referred to the web version of this article.)

An aliquot of the eluted peak material was subjected to semi-preparative RP-HPLC (Fig. 3A), yielding a series of peaks (labeled 1 to 5 according to retention time), which were collected and analyzed by mass spectrometry. The peak with the longest retention time (Peak 1 in Fig. 3A) had a mass corresponding to intact, folded 5P12-RANTES (obs = 7904.7 ± 0.08, expected 7904.8). Its retention time was consistent with that of a chemically synthesized reference standard sample of 5P12-RANTES loaded on the same column on the same day (red trace in Fig. 3A). Both Peak 2 and Peak 3 (the major peak in the chromatogram), had indistinguishable masses (7921.6 ± 0.27), potentially corresponding to either (i) intact folded 5P12-RANTES in which the N-terminal glutamine residue had not yet undergone cyclization to pyroglutamate (expected = 7921.7), or (ii) intact folded 5P12-RANTES in which one of the two methionine residues had been oxidized to Met(O) (expected = 7920.7). Peak 4 had a mass of 7937.6 ± 0.15, consistent with either (i) intact folded 5P12-RANTES in which the N-terminal glutamine residue had not yet undergone cyclization to pyroglutamate and one of the two methionine residues had been oxidized to Met(O) (expected = 7937.7), (ii) intact folded 5P12-RANTES in which the cyclization of the N-terminal glutamine residue had occurred and both methionine residues had been oxidized to Met(O) (expected = 7937.7), or (iii) intact folded 5P12-RANTES in which the cyclization of the N-terminal glutamine residue had occurred and one of the two methionine residues had been oxidized to Met(O_2_). We were unable to determine a mass for Peak 5.

Postulating that the major peak (Peak 3 in Fig. 3A) was likely to be 5P12-RANTES with an uncyclized N-terminal glutamine, we took a second aliquot from material purified by cation exchange chromatography and subjected to it conditions previously shown to favor N-terminal glutamine cyclization [23]. The pH of the fraction was increased to 8.5 using triethylamine and then maintained under N_2_ for 64 h at 37 °C prior to semi-preparative RP-HPLC (Fig. 3B). This treatment resulted in significant reduction of the peak with a retention time corresponding to Peak 3 in Fig. 3A and an increase in the peak corresponding to the authentic product, Peak 1 in Fig. 3A. We concluded that major peak from the partially purified material was intact folded 5P12-RANTES with an uncyclized N-terminal glutamine, and that it can be readily converted into authentic product by appropriate treatment. It is likely that the remaining peaks with shorter retention times than the target material correspond to a mixture of (i) residual 5P12-RANTES with an uncyclized N-terminal glutamine residue, as well as (ii) N-terminal glutamine cyclized and uncyclized 5P12-RANTES carrying one or two oxidized methionine residues.

**Fig. 4:**
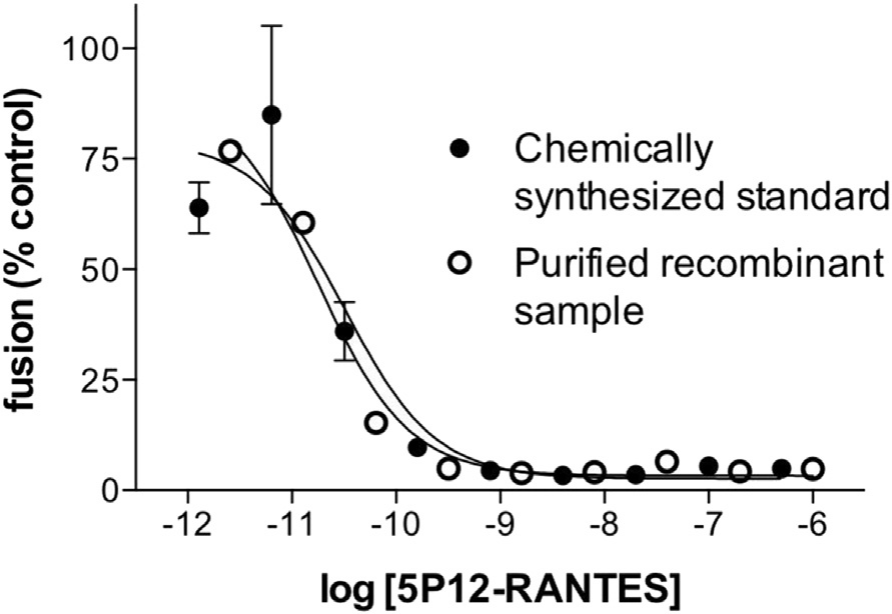
**Anti-HIV activity of the purified recombinant sample of 5P12-RANTES is indistinguishable from that of the chemically synthesized reference standard**. Dilutions of each sample were tested in triplicate for their ability to block R5-tropic HIV envelope-mediated cell fusion in a standard assay [7], [8]. Mean and S.D. are shown for each dilution. Dose-response curves were fitted using Prism (GraphPad).

RP-HPLC-purified material corresponding to Peak 1 in Fig. 3A was tested for biological activity in a CCR5-tropic HIV envelope-dependent cell fusion assay [7], [8] (Fig. 4). The anti-HIV potency of purified sample of recombinant 5P12-RANTES was indistinguishable (IC_50_ 18 pM, 95% confidence interval 8.2–38 pM) from that of the chemically synthesized reference sample (IC_50_ 33 pM, 95% confidence interval 9.4–120 pM).

In summary, without any optimization, pilot production in *P. pastoris* yielded approximately 100 mg/L culture of 5P12-RANTES which could be readily purified and shown to be authentic by RP-HPLC retention time, mass spectrometry and biological activity. Based on these results we opted to proceed with strain optimization and the development of a scaled up cGMP compliant production process to generate clinical grade material.

### 3.3 Scale-up and development of a process for producing cGMP-compliant clinical grade material

#### 3.3.1 Generation of a certified TSE-free parent strain

Since the pilot study was carried under conditions where absence of transmissible spongiform encephalopathy (TSE) elements could not be certified, we opted to generate an entirely new production strain, starting with the *P. pastoris* NRRL Y-11430 strain (ATCC). To ensure no TSE contamination would be passed on from this strain to the production strain, it was sub-cultured through ten serial passages in chemically defined TSE-free certified medium prior to engineering.

#### 3.3.2 Construction of the 5P12-RANTES expression strain

*P. pastoris* NRRL Y-11430 clones transformed with the pPpT4αMF-5P12-RANTES expression vector were isolated and screened for 5P12-RANTES expression. The best expressing clone (clone 17) was selected for scaled-up process development.

#### 3.3.3 Process development strategy

**Fig. 5:**
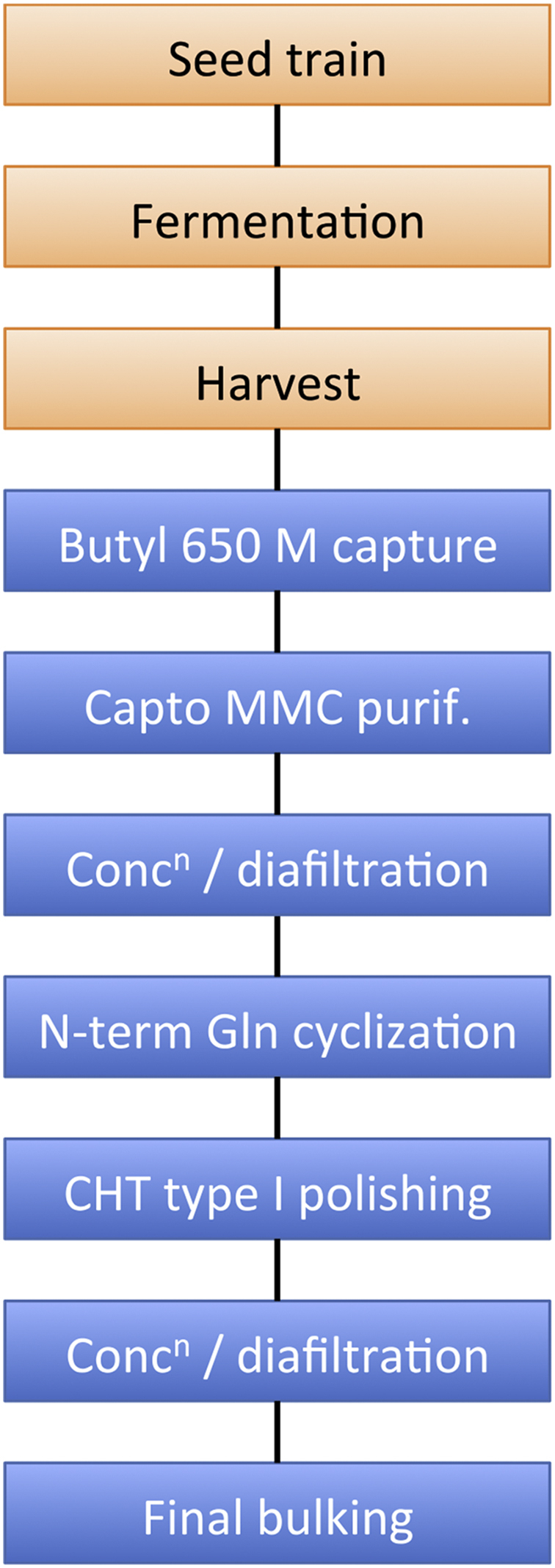
**Outline of scaled-up cGMP-compliant process to produce clinical grade recombinant 5P12-RANTES**. Flow diagram indicating key steps in upstream (orange) and downstream (blue) processes. (For interpretation of the references to colour in this figure legend, the reader is referred to the web version of this article.)

Process development involved optimizing fermentation conditions developing a downstream process that yields clinical grade final product to cGMP specifications. The fermentation procedure does not generate any environmentally harmful contaminants. Firstly, no selection antibiotic is used during the fermentation phase. Secondly, while methanol is used as a sole carbon source during the induction phase of fermentation, its primary metabolite, formaldehyde, does not accumulate as it is further metabolized by the culture [24]. Care was taken in the design of the downstream process to avoid steps that either (i) require expensive solvents or resins, or (ii) would not be readily scalable. The overall process is outlined in Fig. 5. Conditions were initially optimized at the 19 L scale, and then scaled up to the 150 L scale.

#### 3.3.4 Fermentation and harvest

Fermentation conditions were optimized for each stage of the fermentation according to the following parameters: temperature, dissolved oxygen levels, pH, carbon source feed rates. The optimized fermentation protocol of 5P12-RANTES producer strain Clone 17 at the 19 L scale, generated 419 g/L wet weight of cells and 1.60 g/L 5P12-RANTES in a total of 10.9 L culture supernatant. Two 150 L scale runs were performed, a shakedown run and a cGMP run. The shakedown run generated 315 g/L wet weight of cells providing 0.91 g/L 5P12-RANTES in a total of 91.8 L culture supernatant, and the cGMP run generated 218 g/L wet weight of cells, providing and 0.82 g/L 5P12-RANTES in a total of 100.4 L culture supernatant. The lower yields of wet cell weight and volumetric productivity of 5P12-RANTES compared to the 19 L scale fermentation are due adoption of an induction fermentation time of 72 h rather than 96 h. This change was introduced because significant levels of degradation products began to accumulate after 72 h under the fermentation conditions used at the 150 L scale.

#### 3.3.5 Downstream processing

Downstream processing at the 19 L scale following the steps outlined in Fig. 5 provided 3.5 g 96.7% pure 5P12-RANTES, representing an overall yield of 33%. At the 150 L scale, downstream processing provided 24.7 g of 96% pure 5P12-RANTES (overall yield of 28.8%) in the shakedown run, and 27.9 g of 96% pure 5P12-RANTES (overall yield of 30.9%) in the cGMP run. The purification yields obtained at each key step are shown in Table 3, Table 4, Table 5.

### 3.4 The cGMP compliant process yields clinical grade material

**Fig. 6:**
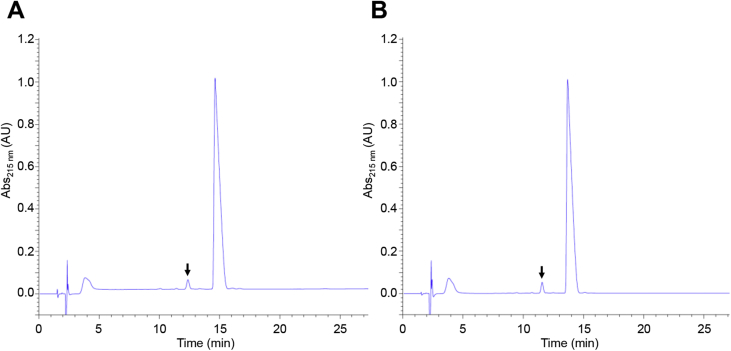
**Purity of 5P12-RANTES produced at 150 L scale after downstream processing**. RP-HPLC chromatogram traces for final material obtained in the shakedown run (A) and the cGMP run (B). The major contaminant peak in each run is indicated with an arrow.

The 19 L engineering run and the two scaled-up processes yielded material that meets standards for clinical grade recombinant protein. A summary of the criteria tested and the results obtained are shown in Table 6. Final RP-HPLC (purity) profiles for each run are highly comparable (Fig. 6). Intact mass spectrometric analysis (unpublished results) indicated that all the detectable contaminants are 5P12-RANTES congeners (peak areas ranging from 0.2% to 2.3%). As expected from the lab scale purification work, these congeners included permutations carrying one or two oxidized methionine residues and residual uncyclized N-terminal glutamine. In addition, we identified a deamidated species and a truncated variant lacking the C-terminal serine residue of the protein. The major contaminating peak corresponds to intact, N-terminal glutamine-cyclized 5P12-RANTES carrying a single oxidized methionine residue.

## 4 Discussion and conclusions

### 4.1 An unusual challenge for manufacturing a biopharmaceutical product

Like many biologics, 5P12-RANTES has an *in vitro* potency in the low pM range [7], but unlike most its mode of administration is by topical application of a gel formulation to genital and other mucosal surfaces. It has been established for a number of topically applied inhibitors that block HIV at or soon after the entry stages that full efficacy *in vivo* requires concentrations many orders of magnitude higher than the *in vitro* IC_50_ [5], [25], [26], [27], [28]. We therefore chose 8 mg/mL (approx. 1 mM, close to the solubility limit of the protein) as the working concentration of 5P12-RANTES for topical use [11]. The conventional dose volume for such indications is 4 mL of gel, i.e. 32 mg/dose per protected sex act. Assuming an average of 80 sex acts per person per year [29], the annual requirement per person would be approximately 2.5 g, i.e. 2.5 tonnes per million people. While the economics of the development and sale of biologics has been shaped by historical and market forces acting on injectables, for topical agents, production could in principle be adapted to the multi-tonne scale and the low-cost constraints more familiar in the food and detergent industries.

### 4.2 Our procedure has the potential to meet manufacturing requirements

*P. pastoris* is a well-established expression platform used in the production of both biopharmaceuticals and industrial proteins [30], [31]. The *P. pastoris* approach that we present represents a promising first step towards levels of low-cost, high yield production that would be required to meet the worldwide need for use in topical HIV prevention. All stages of the process conform to the requirement that they should be scalable, not consume prohibitive quantities of highly expensive materials, nor generate waste products that carry an environmental hazard. Here we obtained multi-gram quantities of cGMP-compliant clinical grade material from a 150 L fermentation, which is more than sufficient for a phase 1 trial. Significant economies of scale will be achievable, and there is scope for the enhancement of volumetric productivity of the producer strain, for example by optimizing fermentation conditions to avoid the accumulation of degradation products that occurred after 72 h at the 150 L scale, increasing transgene copy number [32], co-expressing chaperones to facilitate protein folding and secretion [33], or using a combination of the increased copy number and chaperone co-expression approaches [34].

### 4.3 Properties of 5P12-RANTES in relation to production and purification

Several previous studies have shown that chemokines are readily produced by secretory expression in yeast [14], [15], [16], [17], [18], and in this study we provide another example with 5P12-RANTES. We took advantage of two particular properties of 5P12-RANTES in the chosen process: its high temperature stability made it possible to carry out the cyclization of the N-terminal glutamine residue at 60 °C and its high pI was exploited to facilitate downstream processing. The N-terminal cyclization is an unusual step to find in a downstream processing scheme but is mandated by the fact that the anti-HIV potency of this class of proteins depends on the absence of a positive charge at the N-terminus [6], [7], [8].

### 4.4 A step towards improving access to biologics

In addition to 5P12-RANTES, several other protein-based drugs are being considered for development as topical HIV prevention agents [35], [36], and the economic necessities involved have provided an incentive for researchers to explore methodologies to produce biologics at lower cost, either by adopting a low cost microbial fermentation approach as in this study, or through optimized production in transgenic plants [36], [37], [38]. Progress made in these endeavors has the potential be of benefit not only to those at risk of HIV/AIDS, but also to other patient populations in resource poor settings that currently lack access to biologics. Greater equity of access to biologics worldwide is a declared goal of the United Nations' World Health Assembly [39].

## Figures and Tables

**Table 1:** Gradient program used for analytical RP-HPLC of 5P12-RANTES samples.

Time (min)	% Mobile phase A	% Mobile phase B	% Mobile phase C
0.01	83.3	16.7	0.0
0.50	83.3	16.7	0.0
1.50	10.0	90.0	0.0
4.50	10.0	90.0	0.0
25.43	0.0	100.0	0.0
26.00	0.0	0.0	100.0
29.00	0.0	0.0	100.0
29.50	83.3	16.7	0.0
35.00	83.3	16.7	0.0

**Table 2:** Ten-step methanol feed profile used in scaled-up fermentation process.

Step	Induction time (h)	Feed rate (mL/L/min)	Step	Induction time (h)	Feed rate (mL/L/min)
1	3.0	0.066	6	53.40	0.179
2	13.08	0.080	7	63.48	0.218
3	23.16	0.098	8	73.56	0.267
4	33.24	0.120	9	83.64	0.326
5	43.32	0.146	10	93.72	0.399

**Table 3:** Yields recovered at each key step of the downstream process for the 19 L scale engineering run.

Step	5P12-RANTES (initial), g	5P12-RANTES (final), g	Step yield (%)	Cumulative yield (%)
Harvest	10.11	9.18	90.8	90.8
Butyl 650 M	9.18	10.9	118	107
Capto MMC	10.9	10.9	100.5	108.0
Conc^n^/diafiltration	10.9	9.47	86.7	93.7
Cyclization (N-term. Gln)	9.47	9.84	104	97.4
CHT type I	8.40	4.98	59.3	49.3
Conc^n^/diafiltration	4.98	3.60	72.2	35.6
Final Bulking	3.55	3.47	97.6	34.3

**Table 4:** **Yields recovered at each key step of the downstream process for the 150 L scale shakedown run**. ND; not determined, NA; Not Available.

Step	5P12-RANTES (initial), g	5P12-RANTES (final), g	Step yield (%)	Cumulative yield (%)
Harvest	83.48	94.60	113.3	113.3
Butyl 650 M	94.60	ND	NA	NA
Capto MMC	ND	91.04	NA	NA
Conc/Diafiltration	91.04	79.96	87.8	95.6
Cyclization (N-term. Gln)	79.96	81.53	102.0	97.7
CHT type I	81.53	29.01	35.6	34.8
Conc/Diafiltration	29.01	25.54	88.0	30.6
Final Bulking	25.27	24.68	97.7	29.6

**Table 5:** **Yields recovered at each key step of the downstream process for the 150 L scale cGMP run**. ND; not determined, NA; Not Available.

Step	5P12-RANTES (initial), g	5P12-RANTES (final), g	Step yield (%)	Cumulative yield (%)
Harvest	82.26	95.65	116.3	116.3
Butyl 650 M	95.65	ND	NA	NA
Capto MMC	ND	94.73	NA	NA
Conc/Diafiltration	94.73	81.70	86.2	99.3
Cyclization (N-term. Gln)	81.70	82.58	101.1	100.4
CHT type I	82.58	31.53	38.2	38.3
Conc/Diafiltration	31.53	27.89	88.5	33.9
Final Bulking	27.89	27.87	99.9	33.9

**Table 6:** **5P12-RANTES produced at 19 L and 150 L meets clinical grade criteria**. A series of release assays were performed on final material. Target results and data obtained for each run are shown.

Criterion	Assay	Target result	Engineering run (19 L)	Shakedown run (150 L)	cGMP run (150 L)
Concentration	SCX-HPLC	8.5 ± 0.3 mg/mL	8.3 mg/mL	8.3 mg/mL	8.6 mg/mL
Purity	RP-HPLC	≥95% purity	97%	96%	96%
Aggregation	SEC-HPLC	>95% monomer	100% monomer	100% monomer	100% monomer
Identity	SDS-PAGE	Migration distance conforms to that of the reference standard	Conforms	Conforms	Conforms
Western blot	Conforms	Conforms	Conforms
Potency	Anti-HIV activity	Observed IC_50_ within 70–130% of reference standard	75%	97.5%	92%
Filter/plate	Bioburden	<10 CFU/mL	2 CFU/mL	0 CFU/mL	0 CFU/mL
Endotoxin	LAL	≤65 EU/mL	33.9 EU/mL	7.96 EU/mL	0.36 EU/mL
Host cell protein	ELISA	Report value	110 ng/mL	200 ng/mL	245 ng/mL
Host cell DNA	qPCR	Report value	ND	1 pg/mg protein	1.5 pg/mg protein
pH	pH	≥3.8, <5.0	4.7	4.8	4.6
Conductivity	Conductivity	Report value	0.84 mS/cm	0.80 mS/cm	0.85 mS/cm
